# Launching science, society and policy

**DOI:** 10.1098/rsos.210438

**Published:** 2021-03-24

**Authors:** Adrian Smith, Jeremy K. M. Sanders

**Affiliations:** ^1^ The Royal Society, London, UK; ^2^ Yusuf Hamied Department of Chemistry, University of Cambridge, Lensfield Road, Cambridge CB2 1EW, UK

The Covid-19 pandemic has brought the relationship between science, public policy and society into sharp relief. Across the world, governments have scrambled to mobilize the expertise of scientific communities in the fight against the virus, from the very first sequencing of the Covid-19 genome to the roll out of vaccines barely a year later. Scientists from an array of disciplines are at the forefront of global efforts to defeat the virus and scientific advisers are at the heart of policy-decision-making processes. Nor are these closed or ‘elite’ processes: public understanding, cooperation and engagement are critical to the success or failure of public health strategies.

Science and scientists have never operated in a vacuum, of course, and there have always been complex, institutionally structured interactions between scientific disciplines and scientists, and the societies within which they work. The profound impact of climate sciences is one prominent recent example, but history is replete with powerful relationships between science, the state and society. National variation in these relationships is a fruitful area for comparative study, but the global nature of the challenges mankind faces from climate change or zoonotic diseases, for example, reminds us of the importance of intellectual endeavours that cross borders as well as disciplinary boundaries.

In recent years, *Royal Society Open Science* has published several articles that fall into a broad category of papers that may be described as ‘science for policy’ or ‘policy for science’. Those published so far by authors including David Spiegelhalter FRS (https://royalsocietypublishing.org/doi/10.1098/rsos.181870), Goverdhan Mehta FRS (https://royalsocietypublishing.org/doi/10.1098/rsos.190161, https://royalsocietypublishing.org/doi/10.1098/rsos.200554) and Mark Walport FRS (https://royalsocietypublishing.org/doi/10.1098/rsos.172096) tend to be cross-disciplinary in subject or impact and they have been well received by readers. The journal has been able to offer a home for work like this that may be harder to publish elsewhere because it crosses the boundaries of science, society and policy. These papers and others like them have demonstrated that there is an unmet need for a more structured approach to such content, given the limited publishing avenues in this area. They also address the apparent increasing public and government appetite for accessible policy-related science, in areas such as artificial intelligence and epidemiology. In response to this perceived need, the journal is launching a new ‘Science, Society and Policy’ section to provide a venue for researchers to submit their work for publication and an open access outlet for readers, whether policymakers or interested members of the public.

We (A.S., President, The Royal Society, and J.K.M.S., Editor-in-Chief, *Royal Society Open Science*) have appointed an outstanding Advisory Board including Dame Sally Davies, Sir Michael Marmot and Lord David Willetts in the UK, and Sheila Jasanoff and Mona Nemer in North America. The Chair of the Board, Rupert Lewis, is Head of Science Policy at the Royal Society, providing—perhaps for the first time—a direct link between the Society's important policy role and its publications programme. The Subject Editor is Prof. Nick Pearce, Director of The Institute for Policy Research at the University of Bath, and Associate Editors have been appointed from around the world to ensure the section reflects not only the global nature of science but also in the expectation that the section's content will address questions of global importance. The section will have a broad concern with major societal challenges as diverse as public health, socio-economic inequalities, sustainability and biodiversity. We anticipate that articles will range beyond the scientific disciplines of the journal's other sections, either by virtue of an orientation towards public policy and societal interest, and/or by inter- and multi-disciplinarity. Given the breadth of potential topics we hope to be able to address in the section, we will continue to expand the Advisory and Editorial Boards to ensure our diversity and ability to address areas (both geographical and subject discipline) that may be underrepresented—indeed, we welcome expressions of interest from individuals who would like to be involved.

This is an exciting venture for the Society and the Journal, especially given the support of such eminent figures on the Advisory and Editorial Boards. We hope that this new section will encourage authors to submit research papers, perspectives and reviews exploring the intersection of Science, Society and Policy, papers that pose evidence-based policy recommendations, as well as encourage debate about how policy may affect the performance of science, and for the section to become a ‘go to’ venue for such work. We very much look forward to receiving and publishing our first submissions, and encourage our readers to submit research they think suitable for consideration. We look forward to hearing from you.

